# Dysfunctional KEAP1–NRF2 Interaction in Non-Small-Cell Lung Cancer

**DOI:** 10.1371/journal.pmed.0030420

**Published:** 2006-10-03

**Authors:** Anju Singh, Vikas Misra, Rajesh K Thimmulappa, Hannah Lee, Stephen Ames, Mohammad O Hoque, James G Herman, Stephen B Baylin, David Sidransky, Edward Gabrielson, Malcolm V Brock, Shyam Biswal

**Affiliations:** 1 Department of Environmental Health Sciences, Bloomberg School of Public Health, Johns Hopkins University, Baltimore, Maryland, United States of America; 2 Department of Oncology, Sidney Kimmel Comprehensive Cancer Center, Johns Hopkins University, Baltimore, Maryland, United States of America; 3 Department of Otolaryngology—Head and Neck Surgery, Johns Hopkins University, Baltimore, Maryland, United States of America; 4 Division of Pulmonary and Critical Care Medicine, School of Medicine, Johns Hopkins University, Baltimore, Maryland, United States of America; Dana-Farber Cancer Institute, United States of America

## Abstract

**Background:**

Nuclear factor erythroid-2 related factor 2 (NRF2) is a redox-sensitive transcription factor that positively regulates the expression of genes encoding antioxidants, xenobiotic detoxification enzymes, and drug efflux pumps, and confers cytoprotection against oxidative stress and xenobiotics in normal cells. Kelch-like ECH-associated protein 1 (KEAP1) negatively regulates NRF2 activity by targeting it to proteasomal degradation. Increased expression of cellular antioxidants and xenobiotic detoxification enzymes has been implicated in resistance of tumor cells against chemotherapeutic drugs.

**Methods and Findings:**

Here we report a systematic analysis of the *KEAP1* genomic locus in lung cancer patients and cell lines that revealed deletion, insertion, and missense mutations in functionally important domains of *KEAP1* and a very high percentage of loss of heterozygosity at 19p13.2, suggesting that biallelic inactivation of KEAP1 in lung cancer is a common event. Sequencing of *KEAP1* in 12 cell lines and 54 non-small-cell lung cancer (NSCLC) samples revealed somatic mutations in *KEAP1* in a total of six cell lines and ten tumors at a frequency of 50% and 19%, respectively. All the mutations were within highly conserved amino acid residues located in the Kelch or intervening region domain of the KEAP1 protein, suggesting that these mutations would likely abolish KEAP1 repressor activity. Evaluation of loss of heterozygosity at 19p13.2 revealed allelic losses in 61% of the NSCLC cell lines and 41% of the tumor samples. Decreased KEAP1 activity in cancer cells induced greater nuclear accumulation of NRF2, causing enhanced transcriptional induction of antioxidants, xenobiotic metabolism enzymes, and drug efflux pumps.

**Conclusions:**

This is the first study to our knowledge to demonstrate that biallelic inactivation of KEAP1 is a frequent genetic alteration in NSCLC. Loss of KEAP1 function leading to constitutive activation of NRF2-mediated gene expression in cancer suggests that tumor cells manipulate the NRF2 pathway for their survival against chemotherapeutic agents.

## Abstract 2

Biallelic inactivation of*KEAP1*, a frequent genetic alteration in NSCLC, is associated with activation of the NRF2 pathway which leads to expression of genes that contribute to resistance against chemotherapeutic drugs.

## Editors' Summary

**Background.:**

Lung cancer is the most common cause of cancer-related death worldwide. More than 150,000 people in the US alone die every year from this disease, which can be split into two basic types—small cell lung cancer and non-small-cell lung cancer (NSCLC). Four out of five lung cancers are NSCLCs, but both types are mainly caused by smoking. Exposure to chemicals in smoke produces changes (or mutations) in the genetic material of the cells lining the lungs that cause the cells to grow uncontrollably and to move around the body. In more than half the people who develop NSCLC, the cancer has spread out of the lungs before it is diagnosed, and therefore can't be removed surgically. Stage IV NSCLC, as this is known, is usually treated with chemotherapy—toxic chemicals that kill the fast-growing cancer cells. However, only 2% of people with stage IV NSCLC are still alive two years after their diagnosis, mainly because their cancer cells become resistant to chemotherapy. They do this by making proteins that destroy cancer drugs (detoxification enzymes) or that pump them out of cells (efflux pumps) and by making antioxidants, chemicals that protect cells against the oxidative damage caused by many chemotherapy agents.

**Why Was This Study Done?:**

To improve the outlook for patients with lung cancer, researchers need to discover exactly how cancer cells become resistant to chemotherapy drugs. Detoxification enzymes, efflux pumps, and antioxidants normally protect cells from environmental toxins and from oxidants produced by the chemical processes of life. Their production is regulated by nuclear factor erythroid-2 related factor 2 (NRF2). The activity of this transcription factor (a protein that controls the expression of other proteins) is controlled by the protein Kelch-like ECH-associated protein 1 (KEAP1). KEAP1 holds NRF2 in the cytoplasm of the cell (the cytoplasm surrounds the cell's nucleus, where the genetic material is stored) when no oxidants are present and targets it for destruction. When oxidants are present, KEAP1 no longer interacts with NRF2, which moves into the nucleus and induces the expression of the proteins that protect the cell against oxidants and toxins. In this study, the researchers investigated whether changes in KEAP1 might underlie the drug resistance seen in lung cancer.

**What Did the Researchers Do and Find?:**

The researchers looked carefully at the gene encoding KEAP1 in tissue taken from lung tumors and in several lung cancer cell lines—tumor cells that have been grown in a laboratory. They found mutations in parts of KEAP1 known to be important for its function in half the cell lines and a fifth of the tumor samples. They also found that about half of the samples had lost part of one copy of the *KEAP1* gene—cells usually have two copies of each gene. Five of the six tumors with *KEAP1* mutations had also lost one copy of *KEAP1*—geneticists call this biallelic inactivation. This means that these tumors should have no functional KEAP1. When the researchers checked this by staining the tumors for NRF2, they found that the tumor cells had more NRF2 than normal cells and that it accumulated in the nucleus. In addition, the tumor cells made more detoxification enzymes, efflux proteins, and antioxidants than normal cells. Finally, the researchers showed that lung cancer cells with *KEAP1* mutations were more resistant to chemotherapy drugs than normal lung cells were.

**What Do These Findings Mean?:**

These results indicate that biallelic inactivation of *KEAP1* is a frequent genetic alteration in NSCLC and suggest that the loss of KEAP1 activity is one way that lung tumors can increase their NRF2 activity and develop resistance to chemotherapeutic drugs. More lung cancer samples need to be examined to confirm this result, and similar studies need to be done in other cancers to see whether loss of KEAP1 activity is a common mechanism by which tumors become resistant to chemotherapy. If such studies confirm that high NRF2 activity (either through mutation or by some other route) is often associated with a poor tumor response to chemotherapy, then the development of NRF2 inhibitors might help to improve treatment outcomes in patients with chemotherapy-resistant tumors.

**Additional Information.:**

Please access these Web sites via the online version of this summary at http://dx.doi.org/10.1371/journal.pmed.0030420.

- US National Cancer Institute information on lung cancer and on cancer treatment
- MedlinePlus entries on small cell lung cancer and NSCLC Cancer Research UK information on lung cancer
- Wikipedia entries on lung cancer and chemotherapy (note that Wikipedia is a free online encyclopedia that anyone can edit)

## Introduction

Lung carcinomas are the leading cause of cancer deaths in the United States and worldwide in both men and women. Non-small-cell lung cancer (NSCLC) accounts for approximately 85% of lung cancer cases, with 65% of these patients presenting with unresectable stage III and IV disease [1]. Chemotherapy for advanced, inoperable NSCLC is generally palliative. The major factor contributing to the failure of chemotherapy in lung cancer is the development of drug resistance [2]. Several studies have shown that the expression of xenobiotic metabolism genes (glutathione-*S*-transferases [GSTs]), antioxidants (glutathione [GSH]), and drug efflux proteins (multidrug resistance protein [MRP] family) is increased in NSCLC [3–6]. Xenobiotic metabolism enzymes in conjunction with drug efflux proteins act to detoxify cancer drugs, whereas antioxidants confer cytoprotection by attenuating drug-induced oxidative stress and apoptosis.

Nuclear factor erythroid-2 related factor 2 (NRF2), a cap 'n' collar basic leucine zipper transcription factor, regulates a transcriptional program that maintains cellular redox homeostasis and protects cells from oxidative insult, including from chemotherapeutic agents [7–9]. NRF2 activates transcription of its target genes through binding specifically to the antioxidant response element (ARE) found in those gene promoters. The NRF2-regulated transcriptional program includes a broad spectrum of genes, including ones encoding antioxidants (e.g., γ-glutamyl cysteine synthetase modifier subunit [GCLm], γ-glutamyl cysteine synthetase catalytic subunit [GCLc], heme oxygenase-1, superoxide dismutase, glutathione reductase [GSR], glutathione peroxidase, thioredoxin, thioredoxin reductase, peroxiredoxins[PRDX], and cysteine/glutamate transporter [SLC7A11]) [7,8], xenobiotic metabolism enzymes (e.g., NADP[H] quinone oxidoreductase 1 [NQO1], GSTs, and UDP-glucuronosyltransferase) [7,8], and several ATP-dependent drug efflux pumps (e.g., MRP1 and MRP2) [10–12].

Disruption of *NRF2* in mice enhances oxidative damage and inflammation in lungs on exposure to cigarette smoke [7], diesel exhaust [13], lipopolysaccharide [14], and allergen [15]. *Nrf2*-deficient mice have lesser expression of xenobiotic enzymes and are greatly predisposed to tumors induced by carcinogens such as benzo(a)pyrene and aflatoxin [16]. By virtue of its central role in the transactivation of detoxification genes involved in the cellular responses to oxidative or electrophilic stresses, NRF2 activation is being targeted for cancer chemoprevention [17]. Recent studies have also demonstrated that NRF2 protects against apoptosis induced by oxidants and also by FAS ligand [7,18]. Phosphorylation of NRF2 by protein kinase(s) associated with the MAPK/ERK signaling cascade increases stabilization of NRF2 and has been proposed as an important mechanism leading to increased expression of ARE-dependent genes [11].

**Figure 1:**
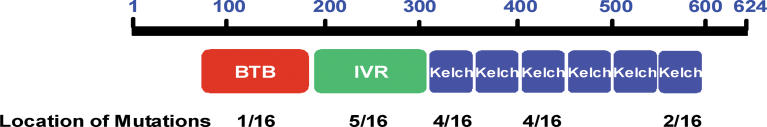
Schematic of the Conserved Domain Structure of Human KEAP1 Protein The protein consists of an N-terminal region (amino acids 1–60), a BTB domain (amino acids 61–179), a central IVR (amino acids 180–314), a double-glycine-rich region comprising six Kelch motifs (amino acids 315–359, 361–410, 412–457, 459–504, 506–551, and 553–598), and a C-terminal domain (amino acids 599–624). The frequency of mutations detected within each domain is indicated below. Amino acid positions of the mutations are listed in Table 1.

Kelch-like ECH-associated protein 1 (KEAP1) is a cytoplasmic anchor of NRF2 that also functions as a substrate adaptor protein for a Cul3-dependent E3 ubiquitin ligase complex to maintain steady-state levels of NRF2- and NRF2-dependent transcription [19,20]. *KEAP1* is located at 19p13.2 and has three major domains: an N-terminal broad complex, tramtrack, and bric-a-brac (BTB) domain; a central intervening region (IVR); and a series of six C-terminal Kelch repeats [21] (Figure 1). The Kelch repeats of KEAP1 bind the Neh2 domain of NRF2, whereas the IVR and BTB domains are required for the redox-sensitive regulation of NRF2 through a series of reactive cysteines present throughout this region [22]. KEAP1 constitutively suppresses NRF2 activity in the absence of stress; however, oxidants, xenobiotics, and electrophiles hamper the KEAP1-mediated proteasomal degradation of NRF2, which results in increased nuclear accumulation and, in turn, the transcriptional induction of target genes that ensure cell survival. [7,23] Recently, Padmanabhan et al. [24] reported a heterozygous somatic mutation (G430C) in *KEAP1* in one lung cancer tumor and a G364C mutation in an adenocarcinoma and a small cell lung cancer cell line.

**Table 1:**
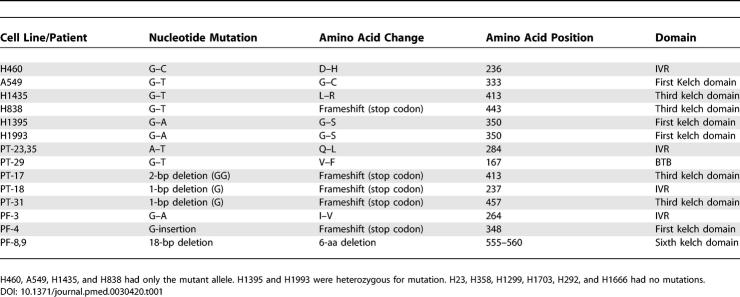
KEAP1 Sequence Alterations in Lung-Tumor-Derived Cell Lines and Patients

To investigate the NRF2–KEAP1 pathway in lung cancer we first examined the status of the *KEAP1* locus at 19p13.2. We also sequenced *KEAP1* in 12 cell lines and 56 tumor samples obtained from lung cancer patients. Since KEAP1 is an inhibitor of NRF2-mediated gene expression, we monitored the expression of NRF2 and its downstream target genes in lung cancer samples.

## Methods

### Tumor Samples

Tumors for this study were randomly selected from 700 freshly frozen lung cancer tissues from patients who had undergone complete, curative resection of their disease from 1993 to 2001 at Johns Hopkins Hospital, Baltimore, Maryland, United States. Tumors were macrodissected and only those specimens with greater than 50% neoplastic cells were used. A total of 56 cases of lung tumor, including 40 paired lung tumors and adjacent normal tissues (frozen tissue) and 16 pleural fluid (PF) samples, were chosen in accordance with the Institutional Review Board protocol, and DNA was isolated using DNeasy Kit (Qiagen, Valencia, California, United States). Details of the lung tumor samples are listed in Table S1.

### PCR and Sequence Analysis

The genomic sequence of human *KEAP1* was downloaded from the NCBI human genome database. Primers for PCR amplification and sequencing of *KEAP1* were designed using Primer3 software (http://fokker.wi.mit.edu) and synthesized by Integrated DNA Technologies (Coralville, Iowa, United States). PCR amplification of DNA from early passage cell lines or primary tumors (PTs) was carried out using ExTaq Premix from Takara Mirus Biosciences (Madison, Wisconsin, United States) and 10 pmol of each primer. PCR products were directly sequenced after purification (QIAquick PCR purification kit, Qiagen). Sequencing was carried out at the DNA sequencing core facility located at Johns Hopkins University. The sequence data were downloaded, assembled, and analyzed to identify potential genetic alterations. All mutations were confirmed by sequencing in both directions. PCR products from samples showing deletion mutation were reamplified using high-fidelity Taq polymerase (Applied Biosystems, Foster City, California, United States) and cloned into TOPO TA cloning vector (Invitrogen, Carlsbad, California, United States), and five clones from each sample were sequenced. All deletions were present in at least two of the five clones sequenced. Exons harboring mutations were reamplified from the original DNA and sequenced. Chromatograms were analyzed by manual review. Sequences of all the primers used for amplification are in Table S2. The PCR cycling conditions were 94 °C (3 min) for one cycle, 94 °C (30 s), 65 °C (45 s), and 72 °C (1 min) for 35 cycles, and a final extension of 72 °C (5 min).

### Microsatellite-Based Loss of Heterozygosity Analysis

The Cancer Genome Project at Sanger Institute (Cambridge, United Kingdom) genotyped 829 cell lines with 395 polymorphic CA/GT repeat markers that provide a 10-cM resolution map of the human genome. The markers are sourced from the ABI Prism LD20 set (data available at http://www.sanger.ac.uk/cgi-bin/genetics/CGP/genotyping/lohmap). Of the 829 cell lines genotyped, 191 were lung cancer cell lines. We obtained the raw data from Sanger Institute and analyzed it, and the results are presented in the form of a heat map. Fluorescent loss of heterozygosity (LOH) analysis using genomic DNA from matched normal and lung tumor tissues was performed using two novel microsatellite markers. Contig AC011461.5.1.100680, containing *KEAP1* flanking sequences, was downloaded from the Ensembl database (http://www.ensembl.org/Homo_sapiens). Two pairs of fluorescently labeled microsatellite primers flanking *KEAP1* (*KEAP-UM1* [CA_17_], present upstream of the *KEAP1* locus, and *KEAP-DM1* [CA_21_], present downstream of the *KEAP1* locus) were designed using Primer3 software and synthesized by Integrated DNA Technologies. Briefly, each PCR reaction was performed in a total volume of 10 μl containing 50 ng DNA, 10 mM Tris-HCl (pH 8.4), 50 mM KCl, 1.5 mM MgCl_2_, 0.01% gelatin, 200 μM dNTPs, 10 pmol of each primer, and 0.1 U Ampli Taq GOLD DNA polymerase (Applied Biosystems). Cycling conditions were 94 °C (10 min) for one cycle, 94 °C (30 s), 66–67 °C (30 s), and 72 °C (30 s) for 25 cycles, and a final extension of 72 °C (20 min), in a MJ Research tetrad PCR System (Bio-Rad, Hercules, California, United States). Details of the primer sequences are listed in Table S2. The data were analyzed by the ABI Genescan and Genotyper software packages (PerkinElmer, Wellesley, Massachusetts, United States), and allelic loss was scored. The sequence and allele size range for *KEAP-DM1* and *KEAP-UM1* were determined prior to their use in the analysis of the normal and tumor-matched lung cancer samples. In our system, a relative allele ratio of less than 0.6, which correlates with an allele loss of approximately 40%, was defined as LOH. The LOH was confirmed at least twice for each marker.

### Heat Maps

Heat maps were constructed using GeneCluster and Treeview software (M. Eisen; http://rana.lbl.gov/EisenSoftware.htm). For displaying gene expression values for the different normal and cancer cell lines, the relative fold changes for individual cell lines were normalized to the maximum fold change value across all cell lines, for a particular gene.

### Western Blot Analysis

To obtain total protein lysates, cancer cells or tissues were lysed in 50 mM Tris (pH 7.2), 1% Triton X-100 containing Halt Protease Inhibitor cocktail (Pierce, Rockford, Illinois, United States) and centrifuged at 12,000*g* for 15 min at 4 °C. To obtain nuclear extracts, NE-PER Nuclear Extraction Reagents (Pierce) were used. Protein concentrations were estimated by the BCA method (Pierce).

For immunoblot analysis, 30 μg and 100 μg of protein from nuclear extracts and total protein lysates, respectively, were used and resolved on 10% SDS-PAGE gels. Proteins were transferred onto PVDF membranes, and the following antibodies were used for immunoblotting: anti-KEAP1 (gift from M. Velichkova**,** University of California San Diego, La Jolla, California, United States), anti-NRF2 (H-300; Santa Cruz Biotechnology, Santa Cruz, California, United States), anti–Lamin B1 (Santa Cruz Biotechnology), and anti-GAPDH (Imgenex, Sorrento Valley, California, United States). All primary antibodies were diluted in PBS-T/5% nonfat dry milk and incubated overnight at 4 °C.

### Enzyme Assay

Enzyme activities of GST, GSR, and NQO1 were determined in the total protein lysates by following methods previously described [8]. Total glutathione (oxidized and reduced) was determined using a modified Tietze method [25] by measuring reduction of 5,5′-dithiobis-2-nitrobenzoic acid in a GSR-coupled assay.

### In Vitro Transcription/Translation

Human *KEAP1* and *NRF2* cDNA was in vitro transcribed and translated using in vitro transcription/translation reactions. These were performed in T7 TNT reticulocyte lysate (Promega, Madison, Wisconsin, United States) following the supplier's instructions.

### Immunohistochemistry

Formalin-fixed tissues were treated with anti-NRF2 antibody (H-300, Santa Cruz Biotechnology) at a dilution of 1:250 for 1 h and developed using horseradish peroxidase (Dako, Glostrup, Denmark). Non-immune rabbit IgG (Jackson ImmunoResearch Laboratories, West Grove, Pennsylvania, United States) was used as a negative control. To demonstrate the specificity of antibody staining, we preincubated the anti-NRF2 antibody with NRF2 and luciferase in vitro transcribed and translated protein, respectively, for 30 min and then carried out immunohistochemical staining.

### Real-Time RT-PCR

Total RNA was extracted from cells using RNeasy kit (Qiagen) and was quantified by UV absorbance spectrophotometry. The reverse transcription reaction was performed by using the Superscript First-Strand Synthesis System (Invitrogen) in a final volume of 20 μl containing 2 μg of total RNA, 100 ng of random hexamers, 1× reverse transcription buffer, 2.5 mM MgCl_2_, 1 mM dNTP, 10 U of RNaseOUT, 20 U of Superscript reverse transcriptase, and DEPC-treated water. Quantitative real-time RT-PCR analyses of human *KEAP1, NRF2, GCLc, GCLm, GSR, PRDX1, GSTA3, GSTA2, NQO1, MRP1,* and *MRP2* were performed by using Assay-on-Demand primers and probe sets from Applied Biosystems. Assays were performed by using the ABI 7000 Taqman system (Applied Biosystems). β-actin was used for normalization.

### Plasmid Construction

Plasmid encoding human *KEAP1* cDNA in pCMV6-XL5 was purchased from Origene Technologies (Rockville, Maryland, United States). Glycine-to-serine mutations, leucine-to-arginine mutations, and a single nucleotide deletion were introduced into the *KEAP1* expression vector by using a site-directed mutagenesis kit (Stratagene, La Jolla, United States). Details of the site-directed mutagenesis primers used in this study are listed in Table S2.

### Cell Culture and Reagents

HBE4, NL20, A549, H460, H1435, H292, H23, H358, H1299, H1993, H1395, and H838 cells were purchased from American Type Culture Collection (Manassas, Virginia, United States) and cultured under recommended conditions. BEAS2B cells were provided by S. Reddy (Johns Hopkins University, Baltimore, Maryland, United States). All transfections were carried out using Lipofectamine 2000 (Invitrogen).

### Generation of Stable Transfectants

H838 cells overexpressing ARE luciferase reporter plasmid were obtained by transfecting H838 cells with 3 μg of *NQO1-ARE* reporter plasmid and 0.3 μg of pUB6 empty vector (Invitrogen). Stable transfectants were selected using Blasticidin at a concentration of 6 μg/ml. Stable clones were expanded and screened for the expression of ARE luciferase.

### Luciferase Assay

H838 cells stably expressing NQO1-ARE luciferase were seeded onto a 24-well dish at a density of 0.2 × 10^6^ cells/ml for 12 h before transfection. WT*-KEAP1* cDNA constructs along with the mutant cDNA constructs (G333C and L413R) were transfected into the cells along with pRL-TK plasmid expressing Renilla luciferase as a transfection control. Twenty-four hours after transfection, cells were lysed and both firefly and Renilla luciferase activities were measured with a Dual-Luciferase Reporter Assay System (Promega).

### siRNA Duplex Screening and Transfection

The siRNA sequence targeting *NRF2* corresponds to the coding region nucleotides 1903–1921 (5′-GTAAGAAGCCAGATGTTAA-3′) in the *NRF2* cDNA. The *NRF2* siRNA duplex with the following sense and antisense sequences was used: 5′-GUAAGAAGCCAGAUGUUAAdUdU-3′ (sense) and 3′-dUdUCAUUCUUCGGUCUACAATT-5′ (antisense). *KEAP1* siRNA corresponds to the coding region nucleotides 1545–1563 (5′-GGGCGTGGCTGTCCTCAAT-3′) in *KEAP1* transcript variant 2. The *KEAP1* siRNA duplex with the following sense and antisense sequences was used: 5′-GGGCGUGGCUGUCCUCAAUdUdU-3′ (sense) and 3′-dUdUCCCGCACCGACAGGAGUUA-5′ (antisense). To confirm the specificity of the inhibition, the siCONTROL non-targeting siRNA 1 (NS siRNA; 5′-UAGCGACUAAACACAUCAAUU-3′) with microarray-defined signature was used as a negative control. All of the siRNA duplexes were synthesized by Dharmacon Research (Lafayette, Colorado, United States). Cells in the exponential growth phase were plated at a density of 0.2 × 10^6^ cells/ml, grown for 12 h, and transfected twice at an interval of 48 h with 50 nM siRNA duplexes using Lipofectamine 2000 and OPTI-MEM reduced serum medium (Invitrogen) according to the manufacturer's recommendations. Concentrations of siRNAs were chosen on the basis of dose–response studies (data not shown).

### MTT Cell Viability Assay

The in vitro drug sensitivity to etoposide and carboplatin was assessed by MTT (3-[4,5-dimethylthiazol-2-yl]-2,5-diphenyl tetrazolium bromide) assay. Cells were plated at a density of 10,000 cells for BEAS2B, 5,000 cells for A549, H460, and H838, and 20,000 cells for H1435 in 96-well plates. They were allowed to recover for 12 h and then exposed to various concentrations of etoposide and carboplatin for 72 h. After 72 h, drug cytotoxicity was evaluated by using a MTT reduction conversion assay (Sigma, Saint Louis, Missouri, United States). Forty microliters of MTT at 5 mg/ml concentration was added to each well, and incubation was continued for 4 h. The formazan crystals resulting from mitochondrial enzymatic activity on MTT substrate were solubilized with 200 μl of dimethyl sulfoxide, and absorbance was measured at 570 nm by using a SpectraMAX microplate reader (Molecular Devices, Sunnyvale, California, United States). Each combination of cell line and drug concentration was set up in eight replicate wells, and the experiment was repeated three times. Cell survival was expressed as absorbance relative to that of untreated controls. Results are presented as means ± standard deviation (SD).

## Results

### Somatic Alterations in *KEAP1* in Lung Cancer Cell Lines

**Figure 2:**
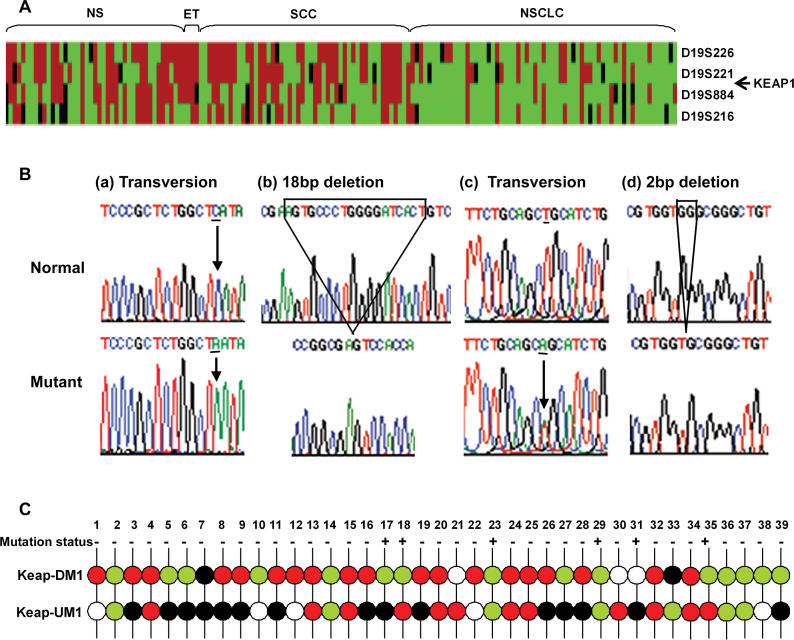
Somatic Alterations in *KEAP1* in Lung Cancer (A) LOH at the 19p13.1–13.3 region. A heat map depicts microsatellite-based LOH at 19p13.1–19p13.3 in 181 lung cancer cell lines. Tumor-derived cell lines that were non-informative for this region were not included. Microsatellite markers showing heterozygous typings are depicted in red, markers demonstrating homozygous typing are in green, and non-informative markers in black. Each vertical column represents one cell line. ET, endocrine tumors; NS, no subtype specified; SCC, small cell carcinoma. (B) Sequence analysis of *KEAP1* mutations in lung cancer. Part a shows the H838 cell line showing C–A substitution (G–T, plus strand), resulting in a termination codon. Wild-type sequence is from BEAS2B. A wild-type allele was not detected in H838. Part b shows a 18-bp deletion in one allele but not in the other allele in the PF DNA sample from PF-8. Part c shows tumor PT-23, showing A–T substitution in one allele but not in the other allele. Part d shows a 2-bp deletion in the fourth exon of *KEAP1* that was detected in one allele of PT-17. Samples showing deletion mutations were confirmed by subcloning and sequencing. (C) LOH at the *KEAP1* locus in human primary lung tumors. Summary of LOH patterns of 39 NSCLC tumors. Retained microsatellites are indicated in red, markers demonstrating allelic loss in green, markers showing genomic instability in white, and non-informative markers in black. *KEAP-UM1* (CA_17_) is present upstream of the *KEAP1* locus, and *KEAP-DM1* (CA_21_) is present downstream of the *KEAP1* locus.

Evaluation of LOH at 19p13.2 in 191 lung cancer cell lines from the Cancer Genome Project database at the Sanger Institute revealed allelic loss in 44% (80/181; ten lines were non-informative) of the cell lines. Of the 72 NSCLC cell lines analyzed in this study, 61% (44/72) showed homozygous typing for at least two consecutive microsatellite markers at 19p13.2 (Figure 2A). To determine whether mutations in *KEAP1* were present in NSCLC, we first amplified and sequenced all five protein-coding exons and intron–exon boundaries of the *KEAP1* gene in a set of 12 lung cancer cell lines. Sequencing of *KEAP1* in these cell lines revealed homozygous mutations in A549, H460, H838, and H1435, whereas H1395 and H1993 were heterozygous for mutant allele (Table 1). We identified five different mutations, among which four were non-conservative amino acid substitutions. A nonsense mutation involving G–T transversion in the fourth exon was detected in the H838 cell line. Representative electropherograms of the mutations are shown in Figures 2B and S1. The microsatellite database at the Sanger Institute showed that the H460, A549, and H838 cell lines had allelic loss (LOH) at 19p13.2. H1435 was not included in the Sanger Institute database. We also sequenced *KEAP1* in four non-malignant (normal) human lung epithelial cell lines (BEAS2B, NL20, HBE4, and NHBE) and found only the wild-type sequence (Table S3).

### Somatic Alterations in *KEAP1* in Lung Cancer Tumor Tissues

To determine whether mutations in *KEAP1* are present in lung cancer tumors, we amplified and sequenced *KEAP1* in 40 primary lung tumors (25 of which were paired with normal tissue samples) and 16 PF samples from lung cancer patients at Johns Hopkins Hospital. The PTs included 19 adenocarcinoma, six large cell carcinoma, and 15 squamous cell carcinoma samples (Table S1). The PF samples included 14 non-small-cell adenocarcinoma and two small cell carcinoma samples (Table S1). Out of 40 PTs sequenced, three tumors (PT-23, PT-29, and PT-35) showed non-conservative amino acid substitution in the IVR and BTB domains of *KEAP1* (Table 1). PT-23 (an adenocarcinoma) and PT-35 (a large cell carcinoma) had identical mutations resulting in Q–L amino acid substitution in the IVR domain (Figure 2B). In addition to this, three PTs (PT-17, PT-18, and PT-31) showed deletion mutation in the IVR and Kelch domains, resulting in frameshift and premature termination codons. Of the 16 PF samples, two samples (PF-8 and PF-9) showed identical 18-bp deletions leading to loss of six amino acids in the sixth Kelch domain. PF-4 had a G-insertion in the third exon resulting in frameshift mutation and truncated KEAP1 protein. I–V substitution was detected in sample PF-3.

To ascertain the status of the *KEAP1* locus in primary lung tumors, we designed two microsatellite markers, *KEAP-UM1* and *KEAP-DM1,* that closely flank the *KEAP1* gene (within ~300 kb on either side). We genotyped the PTs and their corresponding normal DNA samples using these two markers. PF DNA samples were not used for the LOH studies because the corresponding normal DNA was not available. Of the 39 pairs of lung tumors genotyped, 16 tumors (41%) demonstrated LOH for at least one of the markers (Figure 2C). Six tumors showed only microsatellite instability in this region. Five out of six tumors harboring *KEAP1* mutation also demonstrated LOH at these loci. In summary, out of 56 samples sequenced, 54 were NSCLC tumor samples and ten (10/54) of these harbored mutations in *KEAP1*. Interestingly, nine of the ten mutations were identified in adenocarcinomas. Representative electropherograms of the mutations and LOH are shown in Figures S1 and S2.

### Biological Effect of *KEAP1* Mutation in Lung Cancer

**Figure 3:**
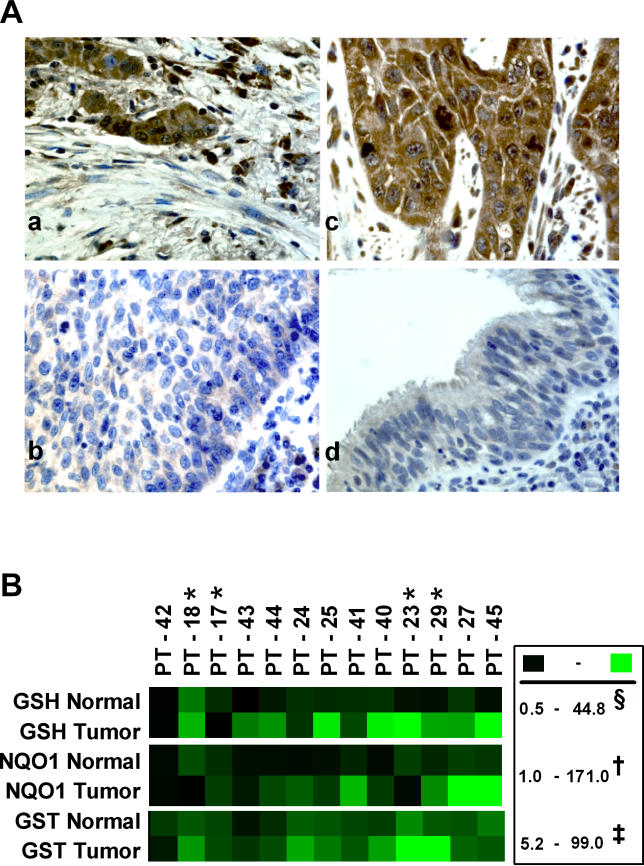
Dysfunctional KEAP1–NRF2 Interaction in NSCLC Tumors (A) Immunohistochemical analysis of NRF2 in NSCLC tissues. Part a shows a patient (PT-18) with mutation in *KEAP1* showing strong nuclear and cytoplasmic staining. Part b shows a patient negative for mutation (PT-28) showing weak cytoplasmic staining. Part c shows a patient negative for mutation (PT-20) showing increased nuclear and cytoplasmic staining in tumor tissue. Part d shows weakly staining normal bronchus from the same patient (PT-20). (B) Total GSH and enzyme activities of NQO1 and total GST in NSCLC and matched normal tissues. Raw data for the heat maps are presented in Table S4. *, samples harboring *KEAP1* mutation; §, nmol/mg protein; †, nmol DCPIP reduced/min/mg protein; ‡, nmol of product formed/min/mg protein.

To study the localization of NRF2 in primary lung tumors, we performed immunohistochemistry on primary lung tumor tissues using anti-NRF2 antibody. Prominent NRF2 staining was detected in both the nucleus and cytoplasm of tumor tissue. All four PT tissues harboring *KEAP1* mutations showed increased NRF2 staining in the nucleus and cytoplasm (Figure 3A, part a). Three tumor tissues (3/5) with wild-type *KEAP1* also demonstrated increased NRF2 staining, whereas the normal bronchus from the same individuals showed very weak staining (Figure 3A, parts c and d). Two tumor tissues with wild-type *KEAP1* showed very weak staining (Figure 3A, part b). We also did immunohistochemistry on five normal bronchi and did not find any significant staining. To confirm the specificity of anti-NRF2 antibody staining, we used in vitro transcribed/translated NRF2 and luciferase (negative control) protein. Incubation of anti-NRF2 antibody with NRF2 protein dramatically reduced the staining, whereas incubation with luciferase protein had no effect on NRF2 antibody staining (Figure S3).

We also studied levels of known NRF2 targets in tumor samples by measuring NQO1 and GST enzyme activities and total GSH levels in 13 pairs of primary NSCLC tumors and adjacent normal tissues. Among these, four pairs (4/13) of samples were from individuals harboring *KEAP1* mutation. Both NQO1 and total GST activities and GSH levels were significantly higher in tumor tissues than in their corresponding normal bronchi (Figure 3B).

**Figure 4:**
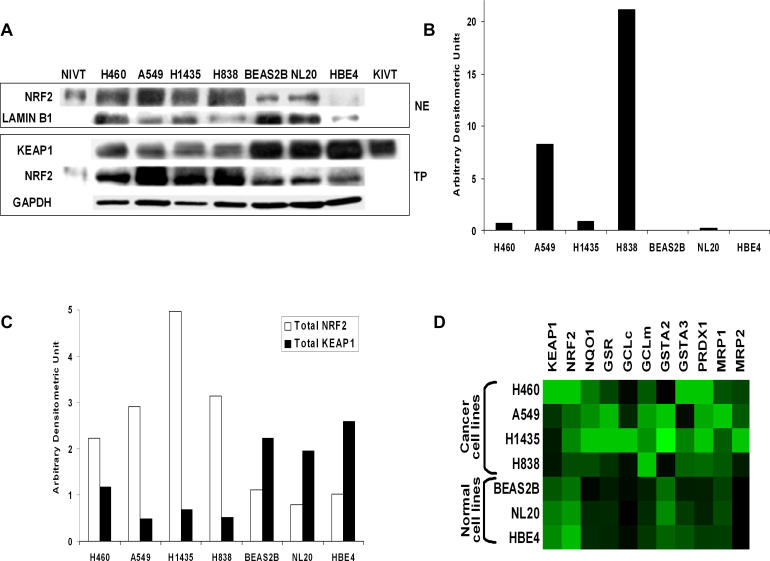
Status of KEAP1 and NRF2 Is Altered in Cancer Cells (A) Immunoblot showing increased nuclear localization of NRF2 in nuclear extracts (NE) from cancer cells. Cancer cells showed lower levels of KEAP1 (~69 kDa) and higher levels of NRF2 (~110 kDa) in total protein lysates (TP). NIVT and KIVT indicate NRF2 and KEAP1 in vitro transcribed/translated product, respectively. (B and C) Quantification of NRF2 and KEAP1 protein in immunoblots. For band densitometry, bands in nuclear extract blot (B) were normalized to Lamin B1, and those in total protein (C) were normalized to GAPDH. (D) Heat map showing relative expression of *KEAP1, NRF2,* and NRF2-dependent genes by real-time RT-PCR. Raw data for the heat maps are presented in Table S5.

**Figure 5:**
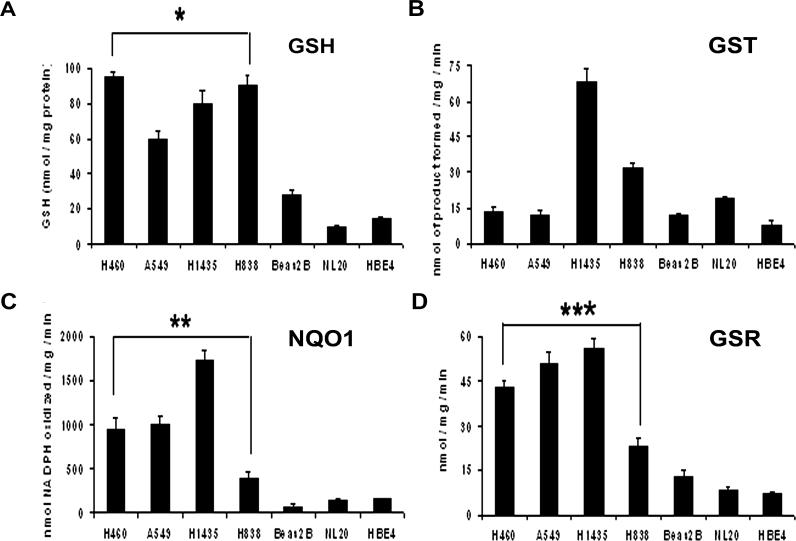
Comparison of Total GSH Levels, GST, NQO1, and GSR Enzyme Activities between Cancer Cells and Normal Cells Shown are total GSH levels (A) and enzyme activities for GST (B), NQO1 (C), and GSR (D). Data represent mean ± SD (*n =* 3). *, *p =* 0.0016; **, *p =* 0.039; ***, *p =* 0.011 relative to normal cells (by *t*-test).

To determine whether *KEAP1* mutations correlated with NRF2 activation, we performed immunoblot analysis to examine the nuclear accumulation of NRF2. For this study, we selected four NSCLC cell lines—H460, A549, H1435, and H838—all harboring KEAP1 mutations, and three non-malignant (normal) cell lines—BEAS2B, NL20, and HBE4. Cancer cells demonstrated increased nuclear localization of NRF2 in comparison with normal cells (Figures 4A–4C and S4). Total NRF2 level was also higher in cancer cells, suggesting that there was increased stabilization of NRF2 in the absence of functional KEAP1. Real-time RT-PCR analysis of *NRF2* and NRF2-dependent target genes revealed that the majority of the NRF2 target genes were upregulated in the cancer cells (Figure 4D). Expression of *MRP2,* known to confer drug resistance, was 100- to 1,000-fold higher in cancer cells. Transcript levels of *NRF2* did not vary considerably between the two groups. As expected, *NQO1* and *GSR* activities and total *GSH* levels were significantly higher in cancer cells (Figure 5). We also analyzed the relative levels of *KEAP1* transcript and protein in cancer cells and normal cells. Importantly, expression of *KEAP1* mRNA was downregulated in three cancer cell lines—A549, H1435, and H838 (Figure 4D)—and KEAP1 protein was downregulated in all four cancer cell lines in comparison with expression in non-malignant cells (Figures 4A–4C and S4).

### Somatic Mutations Reduce KEAP1-Mediated Repression of NRF2

**Figure 6:**
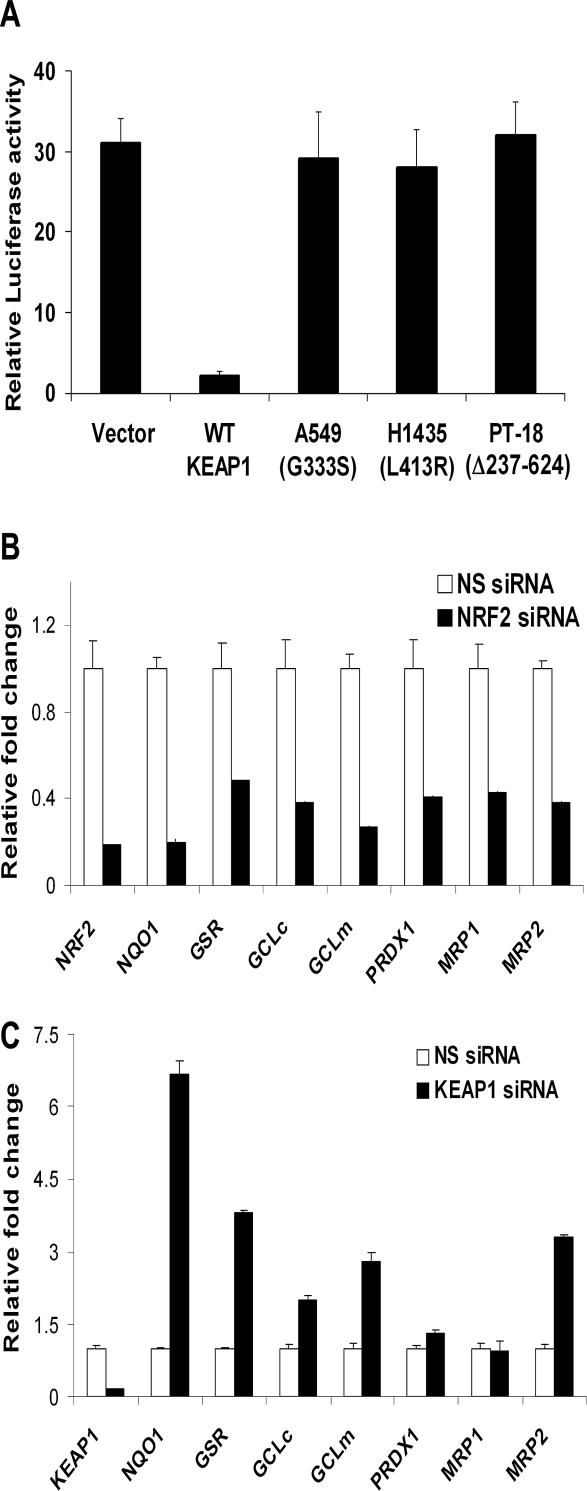
Mutant KEAP1 Protein Is Unable to Suppress NRF2 Activity (A) Repression activity of the KEAP1 mutants was monitored by a luciferase reporter assay. Wild-type and mutant *KEAP1* cDNA constructs were transfected onto H838 cells stably expressing ARE luciferase reporter. Data represent mean ± SD (*n =* 3). (B) Silencing of *NRF2* by siRNA in A549 cells downregulated the expression of NRF2-dependent genes. A nonspecific siRNA (NS siRNA) was used as control. (C) Inhibition of *KEAP1* expression by siRNA in BEAS2B cells upregulated the expression of NRF2-dependent genes.

To determine the functional consequences of tumor-derived mutations on KEAP1 activity and resultant increases in NRF2 activity, we generated cDNAs harboring the same mutations seen in tumor cell lines, A549 (G333C) and H1435 (L413R). We also generated a mutant *KEAP1* construct harboring a single base pair deletion seen in PT-18. We transfected the wild-type and mutant constructs of *KEAP1* into H838 cells stably expressing an ARE reporter. Importantly, all of the three mutants of KEAP1 could not repress the activity of NRF2, whereas overexpression of WT-KEAP1 completely abolished the NRF2-mediated ARE reporter activity (Figure 6A). To further demonstrate that NRF2 activation contributes to the increased expression of antioxidants, xenobiotic metabolism enzymes, and drug efflux pumps in cancer cells, we challenged A549 cells with siRNA targeting *NRF2,* and BEAS2B cells with *KEAP1* siRNA. We measured the transcript levels of *NRF2, NQO1, GSR, GCLc, GCLm, MRP1,* and *MRP2* by real-time RT-PCR. A non-targeting siRNA (NS siRNA) with microarray-defined signature was used as control. Transfection of *NRF2* siRNA in cancer cells decreased the *NRF2* message by 80% and simultaneously downregulated the expression of NRF2 target genes (Figure 6B). Conversely, inhibition of *KEAP1* expression by siRNA induced the expression of NRF2-driven genes in BEAS2B cells (Figure 6C).

### Constitutive Activation of NRF2 Confers Chemoresistance

**Figure 7:**
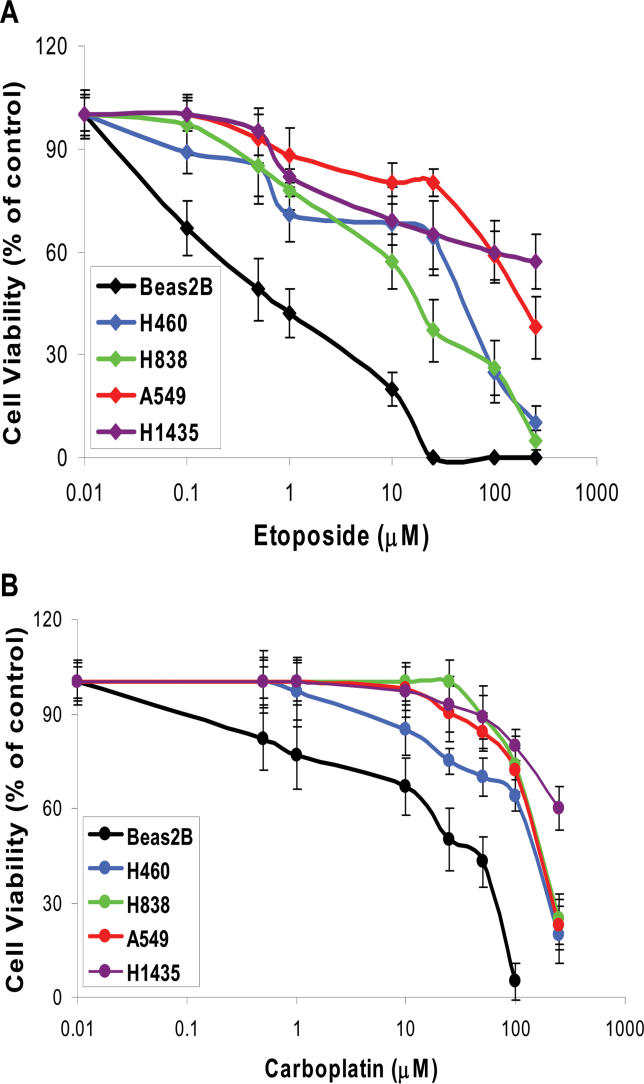
Increased NRF2 Activity Confers Chemoresistance BEAS2B cells and cancer cells were exposed to etoposide (A) or carboplatin (B) for 72 h, and viable cells were determined by MTT assay. BEAS2B cells displayed enhanced sensitivity whereas cancer cells with dysfunctional KEAP1 activity demonstrated reduced chemosensitivity to etoposide and carboplatin treatment. Data are presented as percentage of viable cells relative to the vehicle-treated control. Data are the mean of eight independent replicates, combined to generate the mean ± SD for each concentration.

To elucidate a possible role for NRF2 in conferring chemoresistance, we analyzed the drug resistance profile of normal lung epithelial cells and cancer cells harboring *KEAP1* mutation. Cells were exposed to various concentrations of etoposide and carboplatin, and cell viability was measured after 72 h. As shown in Figure 7, BEAS2B cells with wild-type *KEAP1* demonstrated enhanced sensitivity to both the drugs when compared with that of cancer cells. Reduced chemosensitivity of cancer cells with high NRF2 activity suggests that NRF2 contributes to drug resistance by regulating the expression of several plasma membrane efflux pumps, such as MRP1 and MRP2, and phase II detoxification enzymes.

## Discussion

NRF2, a bZIP transcription factor, activates cellular rescue pathways against oxidative injury, inflammation, apoptosis, and carcinogenesis through transcriptional induction of a broad spectrum of genes involved in xenobiotic detoxification and antioxidant protection [7,8,15]. KEAP1 anchors the NRF2 transcription factor within the cytoplasm, targeting it for ubiquitination and proteasomal degradation, thus repressing its ability to induce cytoprotective genes. LOH at the short arm of Chromosome 19 (19p13.2), where *KEAP1* is located, has been reported in lung cancer [26,27], breast carcinoma [28], prostate cancer [29], oral cancer [30], and gall bladder carcinoma. A genome-wide search for LOH in lung cancer cell lines identified Chromosome 19p as a candidate tumor suppressor region showing more than 60% LOH [26]. Evaluation of LOH at 19p13.2 in lung cancer cell lines from the Cancer Genome Project database at the Sanger Institute revealed allelic losses in 61% of the NSCLC cell lines. We detected point mutations leading to non-conservative amino acid substitutions and nonsense mutations in 50% (6/12) of the cell lines sequenced (Table 1), and all the mutations altered highly conserved amino acid residues located in the Kelch or IVR domain of the KEAP1 protein, suggesting that these mutations would likely abolish KEAP1 repressor activity. Three cell lines—A549, H838, and H1435—displayed G–T transversions, the type of DNA damage expected from bulky DNA adducts caused by the polycyclic hydrocarbons and nitrosoamines in tobacco smoke [31]. Only the mutant allele, and not the wild-type, was detected in the H460, A549, H1435, and H838 cell lines, which suggests that the mutations were either homozygous (which is unlikely) or hemizygous (with corresponding LOH affecting the other allele). The microsatellite database at the Sanger Institute revealed that H460, A549, and H838 cell lines had allelic loss at 19p13.2, which confirms that the wild-type *KEAP1* allele was lost by LOH and that the retained allele was inactivated by somatic mutations. H1435 was not included in the Sanger Institute database.

Sequencing of *KEAP1* in 40 primary lung tumors and 16 PF samples from lung cancer patients revealed deletion, insertion, missense, and frameshift mutations in *KEAP1* in a total of ten tumors, and the frequency of mutations in PTs and PF was 15% (6/40) and 25% (4/16), respectively. Most of the mutations affected highly conserved amino acids located in functionally important domains of KEAP1. We found four different deletions and one single base pair insertion, and all of these alterations (except the 18-bp deletion) introduced a premature stop codon that resulted in a truncated KEAP1 protein. All sequence alterations in this group were heterozygous in tumor DNA and found only in the smokers. Interestingly, the majority (9/10) of these mutations were detected only in adenocarcinomas. No mutations were detected in the paired normal tissue, which confirms that the tumor-derived mutations were somatic in origin. An overall mutation frequency of 19% (10/54) for the *KEAP1* gene in NSCLC tissues suggests that *KEAP1* mutation is a frequent genetic alteration in NSCLC. Microsatellite-based genotyping of PT and paired normal tissues (39 pairs) demonstrated LOH for at least one of the markers in 41.0% (16/39) of the PTs. Five of the six PTs harboring *KEAP1* mutations showed LOH of *KEAP1;* thus, as defined by the Knudson two-hit model, there was biallelic inactivation of *KEAP1* in these tumors [32]. In addition, given the intrinsic difficulties of genetic analysis in PTs, which are invariably contaminated with normal cells, we expect this frequency of mutation and LOH to be an underestimate. Detection of KEAP1 alterations in early stages of lung tumor development (stage IB, Table S1) further suggests that loss of KEAP1 function may be an early event in lung cancer pathogenesis. Future studies aimed at systematic evaluation of KEAP1 status in preinvasive preneoplastic lesions such as hyperplasia, dysplasia, and carcinoma in situ, as well as in late-stage invasive carcinoma, will provide insights regarding the role of the KEAP1–NRF2 pathway in tumor progression.

*Keap1*-null mutation in mice results in postnatal lethality because of constitutive Nrf2 activation, whereas heterozygous mutation of *Keap1* has no effect on Nrf2 activity [23]. This finding suggests that loss of both functional *KEAP1* alleles is essential for constitutive activation of NRF2-mediated gene expression. Thus, a heterozygous mutation in *KEAP1,* as reported recently [24], may not effect NRF2-mediated cytoprotective gene expression. Here, we report—to our knowledge for the first time—biallelic inactivation of KEAP1 in NSCLC, which we believe would result in constitutive activation of NRF*2*.

In corroboration with the above findings that suggest loss of functional KEAP1 in lung cancers, immunohistochemical staining of NRF2 in lung adenocarcinoma tissues showed increased staining in tumor tissue compared to paired normal tissue. As anticipated, NQO1 and GST enzyme activities and GSH levels were significantly elevated in the tumors compared with those in the matched normal tissue. In the absence of functional KEAP1 activity, cancer cells demonstrated an increased nuclear accumulation of NRF2 as well as an increase in total NRF2. Predictably, the NQO1 and GSR enzyme activities and GSH levels were significantly higher in cancer cells. Expression analysis of NRF2-dependent genes revealed upregulation of antioxidant genes, detoxification enzymes, and drug efflux pumps in the cancer cells. GSH and related detoxification enzymes, including GSH-conjugating efflux pumps, are involved in the detoxification of anti-neoplastic drugs and by-products of oxidative stress. Upregulation of GSH and related enzymes in tumor tissues probably contributes to the observed high resistance to cytotoxic drugs and cell death [5].

The high antioxidant capacity of NSCLC cells increases cell survival and proliferation and protects against oxidants, radiation, and chemotherapies, thus conferring the chemoresistance phenotype [3,5]. Levels of GSH-conjugating enzymes have been shown to be higher in cell lines derived from NSCLC cells than in small cell lung cancer cell lines [33]. Others have speculated that these alterations may account for the differences in drug sensitivity between these tumor types [6]. Several studies have documented that GSH concentrations are high in drug-resistant cancer cell lines and that cell viability can be modulated with buthionine sulfoximine, which causes GSH depletion [34,35].

Expression of the wild-type KEAP1 in H838 cells stably expressing an ARE reporter completely abolished ARE reporter activity, whereas overexpression of the KEAP1 mutant construct did not, suggesting that the somatic mutations hamper the association between KEAP1 and NRF2 and consequently activate NRF2. Critically, the enhanced expression of various antioxidants and detoxification genes was verified to be NRF2-dependent, because siRNA targeted against *NRF2* transcript attenuated the expression of these NRF2-dependent genes in A549 cells. However, our understanding of NRF2-regulated genes is based on genomic profiling of Nrf2 wild-type and knock out mice in response to different environmental stress [7,8,15]. Other than the cellular antioxidants and xenobiotic enzymes, the global transcriptional program regulated by constitutive activation of NRF2 in tumor cells remains unknown. Elucidation of NRF2-dependent pathways in tumor cells will provide a better understanding of this novel chemoresistance factor.

Increased NRF2 staining and NRF2-dependent enzyme activities in tumor samples with wild-type KEAP1 suggests that there are other possible mechanisms operating in tumor cells that contribute to increased stabilization and activation of NRF2 [11,36]. For example, Prothymosin α, an extremely abundant nuclear oncoprotein, has been recently demonstrated to be an intranuclear dissociator of NRF2–KEAP1 complex and to upregulate expression of NRF2 target genes [36,37]. Another possible mechanism is differential splicing of *KEAP1,* leading to nonfunctional KEAP1 protein product in cancer cells. Alternatively spliced *KEAP1* transcript lacking exon 5 is reported in human large cell lung carcinoma (NCBI human genome database). Also, *KEAP1* downregulation by promoter methylation cannot be ruled out because the *KEAP1* promoter contains CpG-rich regions. It is also possible that chronic oxidative stress, resulting from increased metabolic activity in cancer cells, is contributing to activation of NRF2. Thus, more intensive studies are needed to unravel the mechanisms responsible for exaggerated NRF2 response in cancer cells even in the presence of wild-type *KEAP1*.

In summary, though activation of the NRF2 pathway in normal cells confers cytoprotection against oxidative stress and carcinogens, unrestrained activation of the same transcriptional program in cancer cells may provide selective growth advantages to tumors over normal somatic cells. The identification of *KEAP1* mutations in a subset of NSCLC samples and the association between loss of functional KEAP1 and increased NRF2 activity extends the emerging paradigm whereby increased expression of antioxidants, detoxification enzymes, and drug transporters favors malignant progression and renders tumors resistant to chemotherapy. While the number of tumors examined in this study is not sufficiently large to lead to a definitive conclusion, this study is the first to our knowledge to show that mutations and LOH in *KEAP1,* as well as possibly other non-mutation-based mechanisms, enable high NRF2 activity in primary lung tumors. It remains to be determined whether such a mechanism operates in cancers arising in other organs such as ovary, pancreas, brain, and breast, where chemoresistance is a major issue, and whether high NRF2 activity in tumors can be a biomarker of poor prognosis. If future experiments verify that enhanced activation of NRF2 is a key cause of chemoresistance and poor prognosis, one could envision development of specific siRNA or small molecule inhibitors of NRF2 for treatment of patients with tumors resistant to chemotherapy.

## Supporting Information

Figure S1 Sequence Analysis Reveals Mutations in *KEAP1* in Lung Cancer Cell Lines and Tumor Tissues (A) Representative DNA sequence data from lung cancer cell lines. The wild-type sequences are from the non-malignant cell line BEAS2B. Only the mutant allele was detected in the cancer cell lines shown.
(B) Representative electropherograms depicting some of the *KEAP1* mutations identified in NSCLC tumors. All the mutations identified in patients were heterozygous.
(122 KB PDF)

Figure S2 LOH at the *KEAP1* Locus in Human Primary Lung Tumors Representative electropherograms showing partial and complete loss of one allele and microsatellite instability at 19p13.2.
(111 KB PPT)

Figure S3 Immunohistochemical Staining of NRF2 in NSCLC Tumor Tissue To demonstrate the specificity of anti-NRF2 antibody staining, we preincubated the anti-NRF2 antibody with luciferase (A) and NRF2 (B) in vitro transcribed and translated protein. Incubation of anti-NRF2 antibody with NRF2 protein significantly reduced the NRF2 staining.
(5.8 MB PPT)

Figure S4 Western Blot Showing Increased Nuclear NRF2 in Lung Cancer Cells(A) Second set of immunoblot showing increased nuclear localization of NRF2 in nuclear extracts (NE) from cancer cells. (B and C) Quantification of NRF2 and KEAP1 protein in immunoblots. For band densitometry, bands in nuclear extract blot (B) were normalized to Lamin B1 and those in total protein (C) were normalized to GAPDH.
(581 KB PPT)

Table S1 Pathological and *KEAP1* Mutation Details of PTs and PF Samples (70 KB PDF)

Table S2 Primer Sequences Used in Study (21 KB XLS)

Table S3 Details of Somatic Alterations in *KEAP1* in Normal and Tumor-Derived Lung Cell Lines (22 KB XLS)

Table S4 Total GSH and Enzyme Activities of NQO1 and GST in Matched NSCLC and Normal Tissues (22 KB XLS)

Table S5 Real-Time Expression Analyses of *KEAP1*, *NRF2*, and *NRF2*-Dependent Genes by Real-Time RT-PCR (18 KB XLS)

### Accession Numbers

The National Center for Biotechnology Information (http://www.ncbi.nlm.nih.gov) accession numbers for the genes and gene products discussed in this paper are *GCLC* (GeneID: 2729), *GCLM* (GeneID: 2730), *GSR* (GeneID: 2936), *KEAP1* (GeneID: 9817), KEAP1 (NP_036421, NP_987096), *MRP1* (GeneID: 4363), *MRP2* (GeneID: 1244), *NQO1* (GeneID: 1728), *NRF2* (GeneID: 4780), NRF2 (NP_006155), and *PRDX1* (GeneID: 5052).

## Abbreviations

ARE: antioxidant response element
BTB: broad complex, tramtrack, and bric-a-brac
GCLc: γ-glutamyl cysteine synthetase catalytic subunit
GCLm: γ-glutamyl cysteine synthetase modifier subunit
GSH: glutathione
GSR: glutathione reductase
GST: glutathione-*S*-transferase
IVR: intervening region
KEAP1: Kelch-like ECH-associated protein 1
LOH: loss of heterozygosity
MRP: multidrug resistance protein
NQO1: NADP(H) quinone oxidoreductase 1
NRF2: nuclear factor erythroid-2 related factor 2
NSCLC: non-small-cell lung cancer
PF: pleural fluid
PRDX: peroxiredoxin
PT: primary tumor
SD: standard deviation
