# Supplementary material for: Dysfunctional KEAP1–NRF2 Interaction in Non-Small-Cell Lung Cancer
Source: PLoS Med. 2006 Oct 3;3(10):e420. doi: 10.1371/journal.pmed.0030420 (PMC1584412; doi:10.1371/journal.pmed.0030420)
Supplement: Supplementary file 1 [file pmed.0030420.sg001.pdf]

Figure-S1

A

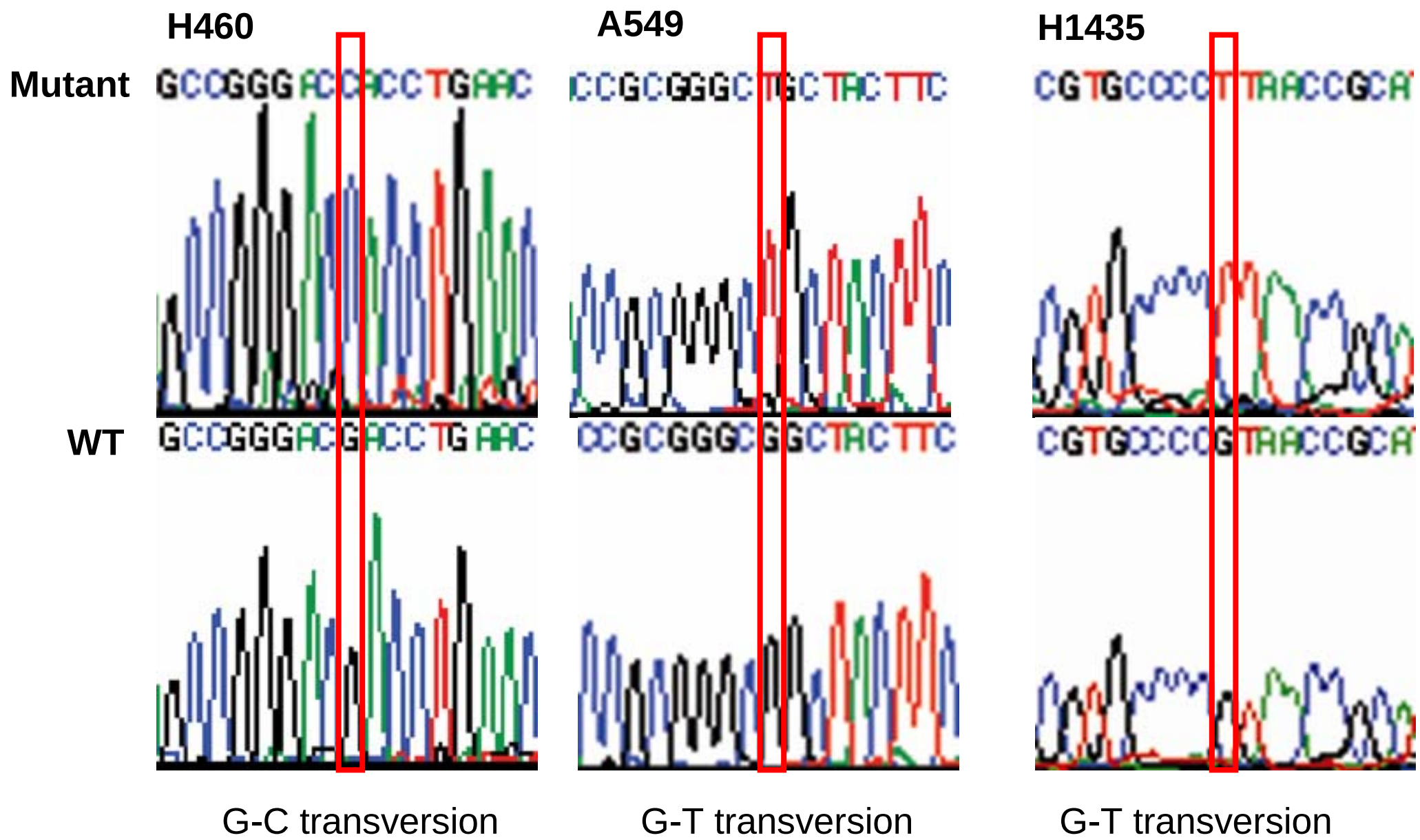
H460
A549
H1435
Mutant
WT
G-C transversion
G-T transversion
G-T transversion

B

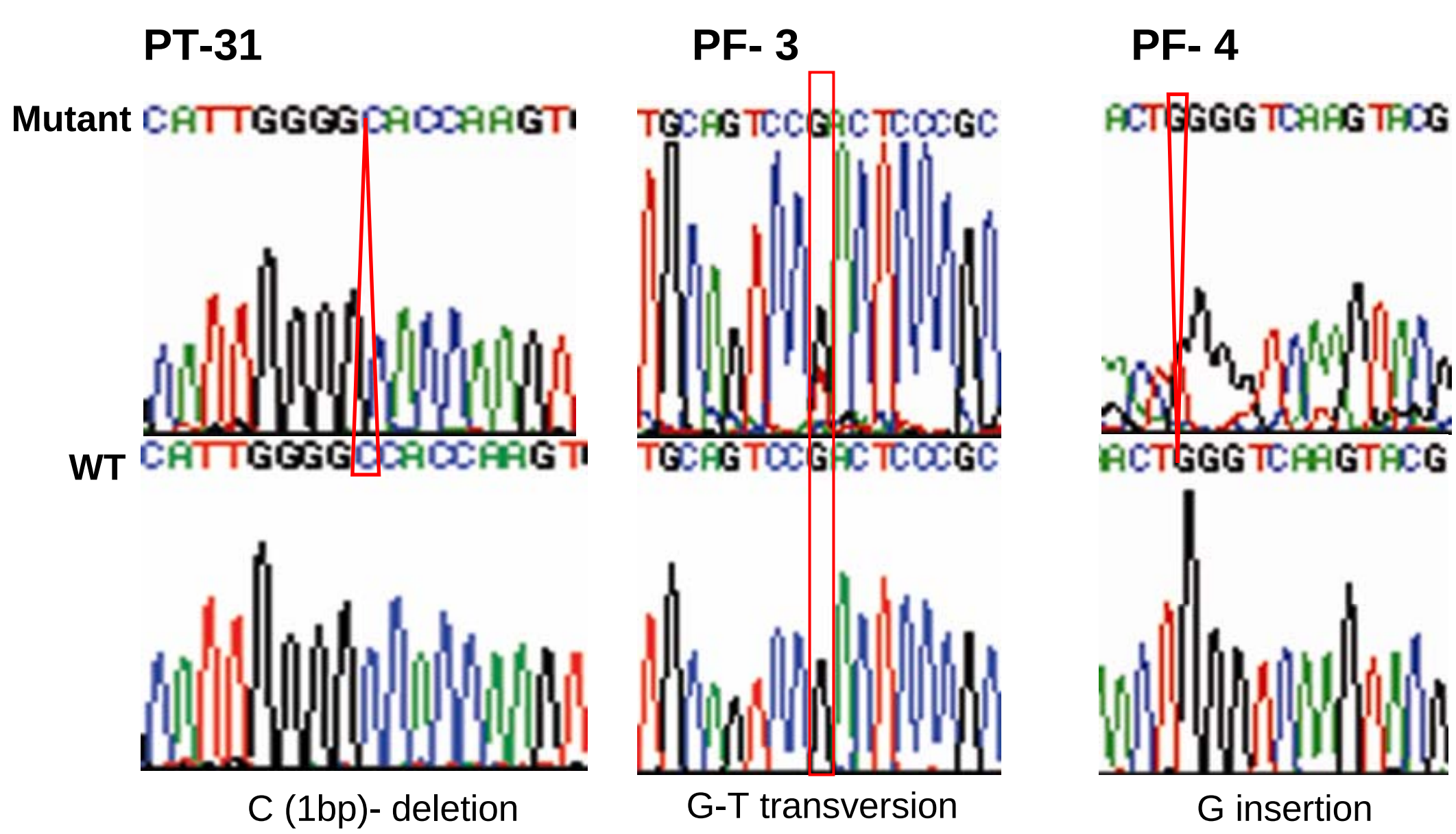
PT-31
PF- 3
PF- 4
Mutant
WT
C (1bp)- deletion
G-T transversion
G insertion
