# Supplementary material for: Dysfunctional KEAP1–NRF2 Interaction in Non-Small-Cell Lung Cancer
Source: PLoS Med. 2006 Oct 3;3(10):e420. doi: 10.1371/journal.pmed.0030420 (PMC1584412; doi:10.1371/journal.pmed.0030420)
Supplement: Supplementary file 2 [file pmed.0030420.st001.pdf]

Table S1A. Pathological and KEAP1 mutation details of

Patient Number	Race	Pathology	Stage	Tumor DNA Sequenced	Normal DNA Sequenced	Mutation Status
**PT-1**	AS	Adenocarcinoma	2B	Yes	Yes	WT
**PT-2**	AA	Adenocarcinoma	1A	Yes	Yes	WT
**PT-3**	CA	Adenocarcinoma	2B	yes	Yes	WT
**PT-4**	CA	Squamous	1A	Yes	Yes	WT
**PT-5**	CA	Squamous	1B	Yes	Yes	WT
**PT-6**	CA	Adenocarcinoma	2B	Yes	Yes	WT
**PT-7**	CA	Adenocarcinoma	2B	Yes	Yes	WT
**PT-8**	CA	Adenocarcinoma	3B	Yes	Yes	WT
**PT-9**	CA	Squamous	1A	Yes	Yes	WT
**PT-10**	AA	Squamous	1B	Yes	Yes	WT
**PT-11**	CA	Squamous	1B	Yes	Yes	WT
**PT-12**	CA	Large Cell	1B	Yes	Yes	WT
**PT-13**	AA	Squamous	1B	Yes	Yes	WT
**PT-14**	AA	Squamous	1A	Yes	Yes	WT
**PT-15**	CA	Squamous	1B	Yes	Yes	WT
**PT-16**	CA	Adenocarcinoma	2B	Yes	Yes	WT
**PT-17**	CA	Adenocarcinoma	3B	Yes	Yes	2 bp deletion
**PT-18**	CA	Adenocarcinoma	1B	Yes	Yes	1 bp deletion
**PT-19**	CA	Squamous	3A	Yes	Yes	WT
**PT-20**	CA	Squamous	3A	Yes	Yes	WT
**PT-21**	CA	Squamous	3B	Yes	Yes	WT
**PT-22**	CA	Squamous	1B	Yes	Yes	WT
**PT-23**	AA	Adenocarcinoma	3A	Yes	Yes	A-T transversion
**PT-24**	CA	Adenocarcinoma	3A	Yes	N.D.	WT
**PT-25**	AA	Squamous	1B	Yes	N.D.	WT
**PT-26**	CA	Large Cell	1B	Yes	N.D.	WT
**PT-27**	CA	Large Cell	1B	Yes	N.D.	WT
**PT-28**	AA	Squamous	1B	Yes	N.D.	WT
**PT-29**	AA	Adenocarcinoma	1B	Yes	N.D.	G-T transversion
**PT-30**	CA	Adenocarcinoma	1B	Yes	N.D.	WT
**PT-31**	CA	Adenocarcinoma	1B	Yes	N.D.	1 bp deletion
**PT-32**	CA	Adenocarcinoma	4	Yes	N.D.	WT
**PT-33**	CA	Adenocarcinoma	2B	Yes	Yes	WT
**PT-34**	CA	Large Cell	1B	Yes	N.D.	WT

**PT-35**	CA	Large Cell	1B	Yes	Yes	A-T transversion
**PT-36**	CA	Adenocarcinoma	3A	Yes	N.D.	WT
**PT-37**	CA	Squamous	1B	Yes	N.D.	WT
**PT-38**	CA	Adenocarcinoma	1A	Yes	N.D.	WT
**PT-39**	CA	Large Cell	1B	Yes	N.D.	WT
**PT-40**	CA	Adenocarcinoma	3A	N.D.	N.D.	N.D.
**PT-41**	CA	Adenocarcinoma	1B	N.D.	N.D.	N.D.
**PT-42**	CA	Squamous	3A	N.D.	N.D.	N.D.
**PT-43**	AA	IMT	4	N.D.	N.D.	N.D.
**PT-44**	CA	Squamous	2B	N.D.	N.D.	N.D.
**PT-45**	CA	Adenocarcinoma	UK	Yes	N.D.	WT

PT, primary tumor; AA, African American; CA, Caucasian; AS, Asian; UK, Unknown; NI, N Determined; aa, amino acid; IMT, Inflammatory myofibroblastic tumor.

Table S1B. Pathological and KEAP1 mutation details of pleural fluid samples.

Patient Nu	Race	Pathology	Stage	PF DNA Seque	Normal DNA Se	Mutation Status
PF-1	CA	Adenocarcinoma	4	Yes	NA	WT
PF-2	AA	Adenocarcinoma	4	Yes	NA	WT
PF-3	CA	Adenocarcinoma	4	Yes	NA	G-A transversion
PF-4	AA	Adenocarcinoma	4	Yes	NA	1 bp Insertion
PF-5	CA	NSCLC	3B	Yes	NA	WT
PF-6	CA	Adenocarcinoma	UK	Yes	NA	WT
PF-7	CA	Adenocarcinoma	4	Yes	NA	WT
PF-8	CA	Adenocarcinoma	3B	Yes	NA	18 bp Deletion
PF-9	CA	Adenocarcinoma	3B	Yes	NA	18 bp Deletion
PF-10	CA	Adenocarcinoma	4	Yes	NA	WT
PF-11	AA	Adenocarcinoma	3B	Yes	NA	WT
PF-12	CA	Adenocarcinoma	4	Yes	NA	WT
PF-13	AA	Adenocarcinoma	4	Yes	NA	WT
PF-14	CA	Small Cell	4	Yes	NA	WT
PF-15	CA	Small Cell	4	Yes	NA	WT
PF-16	CA	Adenocarcinoma	4	Yes	NA	WT

PF, Pleural fluid sample; AA, African American; CA, Caucasian; UK, Unknown; NA, Not availabl

primary tumors.

Amino acid change	LOH status		Smoker
	KEAP-DM1	KEAP-UM1	
None	WT	MIC	Yes
None	LOH	LOH	Yes
None	WT	NI	Yes
None	WT	WT	Yes
None	LOH	NI	No
None	LOH	NI	Yes
None	NI	NI	Yes
None	WT	NI	Yes
None	WT	NI	Yes
None	LOH	MIC	Yes
None	WT	NI	Yes
None	WT	MIC	Yes
None	WT	WT	Yes
None	LOH	LOH	Yes
None	WT	WT	Yes
None	WT	NI	Yes
Stop Codon	LOH	NI	Yes
Stop Codon	LOH	WT	Yes
None	WT	NI	Yes
None	WT	WT	Yes
None	MIC	WT	No
None	WT	MIC	Yes
Q-L	LOH	LOH	Yes
None	WT	WT	Yes
None	WT	WT	Yes
None	WT	NI	Yes
None	LOH	NI	Yes
None	WT	NI	Yes
V-F	LOH	LOH	Yes
None	MIC	WT	Yes
Stop Codon	MIC	NI	Yes
None	WT	WT	No
None	NI	LOH	Yes
None	WT	WT	Yes

Q-L	LOH	WT	Yes
None	LOH	LOH	Yes
None	LOH	LOH	Yes
None	LOH	MIC	Yes
None	LOH	NI	Yes
N.D.	N.D.	N.D.	Yes
N.D.	N.D.	N.D.	Yes
N.D.	N.D.	N.D.	Yes
N.D.	N.D.	N.D.	Yes
N.D.	N.D.	N.D.	Yes
None	N.D.	N.D.	Yes

on-informative, WT, wild type; N.D., Not

Amino acid cha	LOH status		Smoker
	KEAP-DM	KEAP-UM1	
None	NI	NI	No
None	NI	NI	Yes
I-V	NI	NI	Yes
Stop codon	NI	NI	Yes
None	NI	NI	Yes
None	NI	NI	Yes
None	NI	NI	No
6 aa deletion	NI	NI	Yes
6 aa deletion	NI	NI	Yes
None	NI	NI	Yes
None	NI	NI	Yes
None	NI	NI	No
None	NI	NI	UK
None	NI	NI	Yes
None	NI	NI	Yes
None	NI	NI	Yes

e; NI, Non-informative, WT, wild type; aa, amino acid;UK, unknown
